# Natural Product Synthesis: The Endless Quest for Unreachable Perfection

**DOI:** 10.1021/acsorginorgau.3c00040

**Published:** 2023-10-09

**Authors:** Nicolas Fay, Cyrille Kouklovsky, Aurélien de la Torre

**Affiliations:** Institut de Chimie Moléculaire et des Matériaux d’Orsay (ICMMO), Université Paris-Saclay, CNRS, 17 Avenue des Sciences, 91405 Orsay, France

**Keywords:** Total Synthesis, Natural Products, Biosynthesis, Stereoselectivity, Retrosynthesis

## Abstract

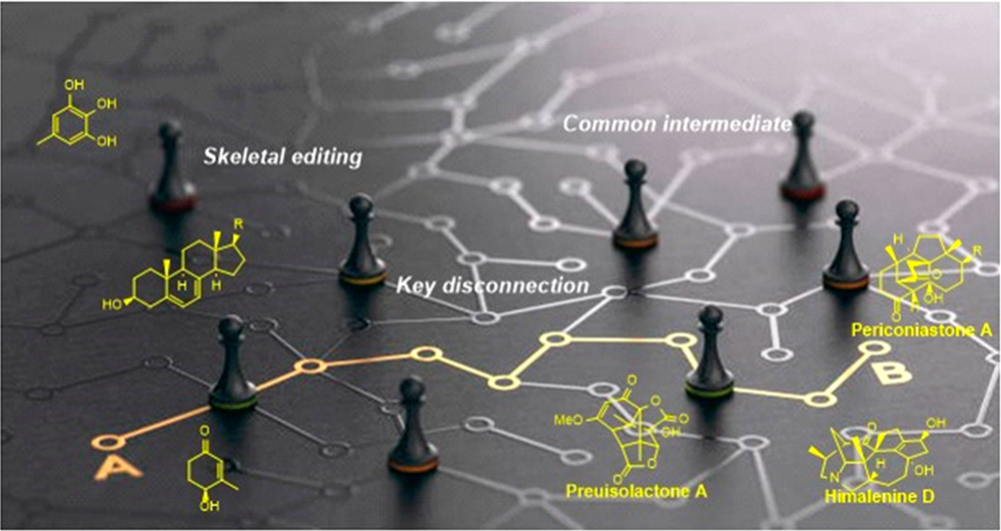

Total synthesis is a field in constant progress. Its practitioners aim to develop ideal synthetic strategies to build complex molecules. As such, they are both a driving force and a showcase of the progress of organic synthesis. In this Perspective, we discuss recent notable total syntheses. The syntheses selected herein are classified according to the key strategic considerations for each approach.

## Introduction

**Scheme 1:**
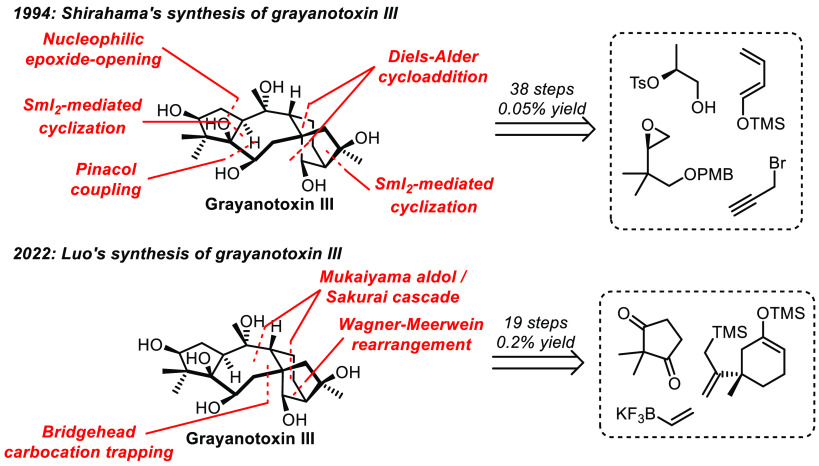
Evolution of Grayanotoxin III Synthesis

Natural product synthesis, the art of building complex molecular architectures using the science of organic chemistry, is often seen as one of the most challenging yet fruitful fields of chemical science. Despite recurrent criticism regarding its supposed decline, it is “*as exciting as ever and here to stay*”, as stated by Baran.^1^ Not only is it the testing ground for new methods and strategies in organic synthesis,^2^ but it also serves the fields of phytochemistry, marine natural products, bacteriology—through structure elucidation^3^—and medicinal chemistry as the first move in many drug discovery campaigns.^4^ Moreover, total synthesis could be considered as a showcase of progress made in the field of organic synthesis. For example, in 1994, the group of Shirahama achieved the first enantioselective synthesis of grayanotoxin III in 38 steps and 0.05% overall yield, a real feat at the time (Scheme 1).^5^ And 28 years later, the group of Luo reported the synthesis of the same molecule in only half the amount of steps and an overall yield 4 times higher (19 steps and 0.2% overall yield).^6,7^ This spectacular improvement was made possible by multiple advances in organic chemistry, especially regarding asymmetric organocatalysis, cascade reactions, and transition-metal catalysis.

The past few years have seen many successes in total synthesis, confirming Baran’s statement. The quest for an ideal synthesis has driven organic chemistry practitioners toward designing efficient and elegant strategies.^8^ The concept of ideality has been defined as the percentage of “productive steps” in the synthetic route, although the same authors clearly indicate that it should not be the sole indicator to judge the quality of a total synthesis.^9^ Indeed, step count, atom and redox economy, scale, or yield are other key indicators of the efficiency and practicability of a synthesis. Moreover, one could argue that the indicators that should be taken into account to judge the quality of a synthesis might also depend on the synthesis’ purpose. In the synthesis of a potential drug candidate, scalability is more important than it is in a synthesis for structure elucidation purposes. In this Perspective, we will try to highlight recent efficient total syntheses. To focus on modern aspects of the field, we limited ourselves to syntheses published in the last 10 years. This collection is very personal and will by no means be exhaustive. The selected syntheses will be classified in three categories depending on the key considerations: (i) divergent syntheses, in which the most important strategic consideration is to identify the right late intermediate; (ii) semisyntheses, in which the key is to remodel the skeleton of an abundant natural product; and (iii) concise syntheses, in which one or two strategic disconnection allow to greatly simplify the retrosynthesis.

## Identifying the Right Common Intermediate: Collective Total Synthesis

The huge diversity of complex natural products that can be considered as interesting targets for total synthesis can look overwhelming. A creative solution to this challenge has been found by using a same strategy to reach various natural products.^10^ Sometimes termed “divergent synthesis”, “collective synthesis” as defined by MacMillan,^11^ or “structure-pattern-based synthesis” as phrased by Gaich,^12^ this approach relies on the identification of a key common intermediate which already bears part of the complexity of a natural product, from which diversity could be accessed in the late stages of the synthesis. These approaches have led to various successes in recent years.

**Scheme 2:**
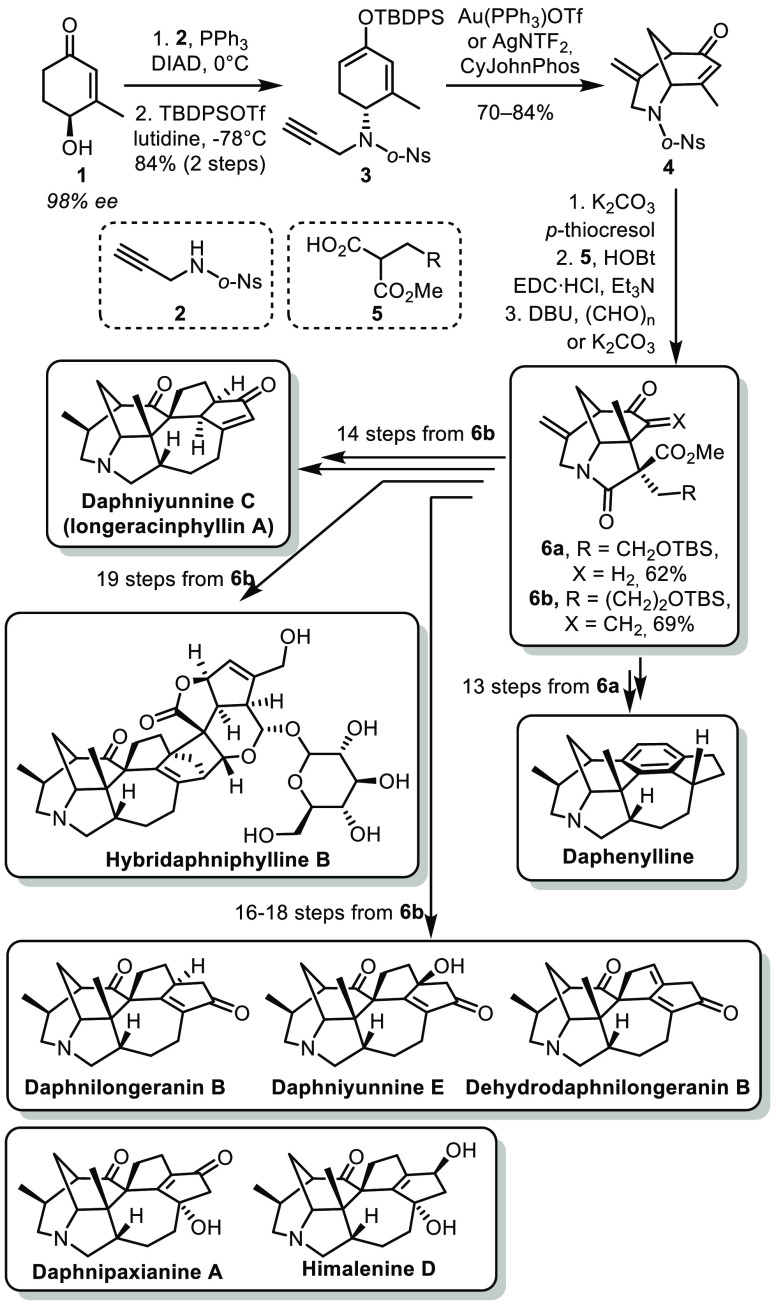
Li’s Divergent Synthesis of *Daphniphyllum* Alkaloids

In the context of a total synthesis campaign targeting the *Daphniphyllum* alkaloids, the group of Li identified a tricyclic structure common to most members of the family as a key intermediate. This could be accessed from known hydroxy enone **1** (easily obtained with high enantiomeric excess in six steps from *m*-methylanisole).^13^ The allylic alcohol was substituted by a propargylamide **2** under Mitsunobu conditions followed by formation of an enol ether **3** (Scheme 2). The bridged piperidine ring **4** was formed through a Au- or Ag-catalyzed Conia-ene reaction. Deprotection of the amine, amide coupling, and intramolecular Michael addition allowed the formation of the fused γ-lactam ring **6a** or **6b**. An important point is the scalability of this synthesis. In all cases, the tricyclic intermediate was obtained on multigram scale, a crucial asset to successfully complete the syntheses. From **6a**, a 13 step sequence allowed the formation of daphenylline.^14^ The key steps were a 6π-electrocyclization/oxidation for the formation of the aromatic ring and Giese addition for the 7-membered ring. On the other hand, a 14 step sequence from **6b** led to the formation of daphniyunnine C.^15^ In this case, the key steps were an intramolecular conjugate addition for the formation of the 7-membered ring, a Lu (3 + 2)-cycloaddition and an intramolecular Horner–Wadsworth–Emmons reaction for the formation of the two fused cyclopentanes. A similar approach was also applied to the synthesis of daphnilongeranin B, daphniyunnine E, dehydrodaphnilongeranin B, and hybridaphniphylline B.^16^ The strategy was later adapted for the synthesis of daphnipaxianine A and himalenine D.^17^

In this work, the identification of an efficient approach for the formation of the tricyclic core common to most *Daphniphyllum* alkaloids was the key to success and allowed the authors to achieve the synthesis of 8 very complex natural products in an impressively efficient way.

**Scheme 3:**
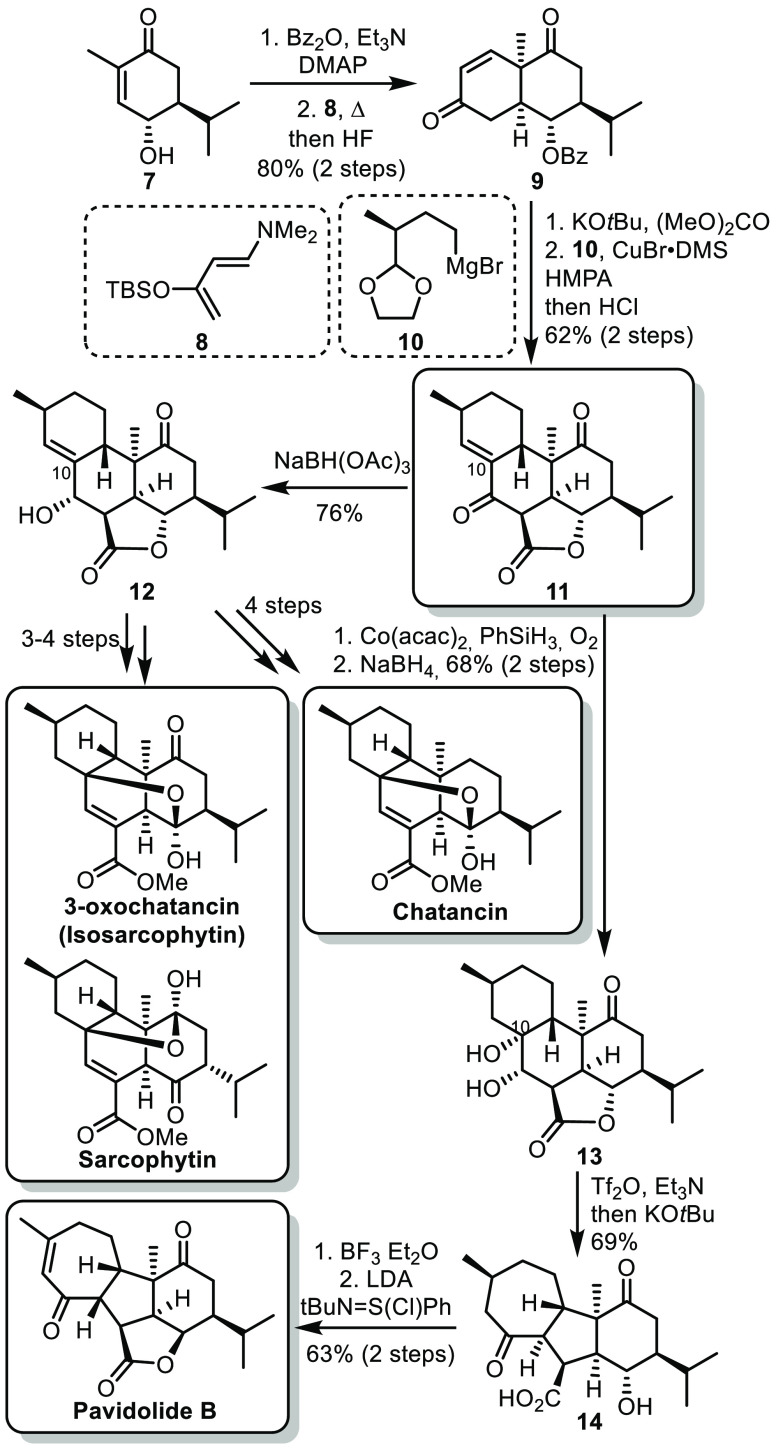
Ding’s Synthesis of Cembranoids from the Sarcophytin Family

In 2019, the group of Ding reported the synthesis of various cembranoids from the sarcophytin family.^18^ The synthesis started from known hydroxy enone **7**, synthesized in two steps from carvone.^19^ The hydroxyl moiety was protected prior to a Diels–Alder cycloaddition with Rawal diene **8** (Scheme 3). The resulting cis-decalin **9** reacted with dimethyl carbonate to form the tricyclic core bearing a γ-lactone, which underwent conjugate addition and *in situ* cyclization under acidic conditions to achieve the fused tetracycle **11**. This became the key intermediate for the synthesis of sarcophytin, chatancin, 3-oxochatancin, and pavidolide B. A chemoselective reduction of the enone **11** led to allylic alcohol **12**, from which a sequence of exhaustive ketone reduction, Mukaiyama hydration, elimination, transesterification, and oxidation led to the formation of chatancin. On the other hand, from **12**, a sequence of Mukaiyama hydration, elimination, and oxidation led to 3-oxochatancin, which could be further epimerized to form sarcophytin. Moreover, this synthesis allowed reassignment of the structure of isosarcophytin as 3-oxochatancin. From **11**, performing the Mukaiyama hydration prior to ketone reduction led to the opposite stereoselectivity at C.^10^ This configuration allowed a semipinacol rearrangement to occur by treatment of **13** with Tf_2_O in basic conditions, affording the tetracyclic core **14**. From there, lactonization and oxidation led to the formation of pavidolide B. Remarkably, the lactonization occurred with inversion of the configuration at the hydroxyl moiety, probably through the formation of a transient cyclopropyl intermediate.

Overall, Ding’s efficient strategy led to the formation of 4 diverse natural products from the sarcophytin family in 10–11 steps (4–5 steps from key intermediate **11**).

**Scheme 4:**
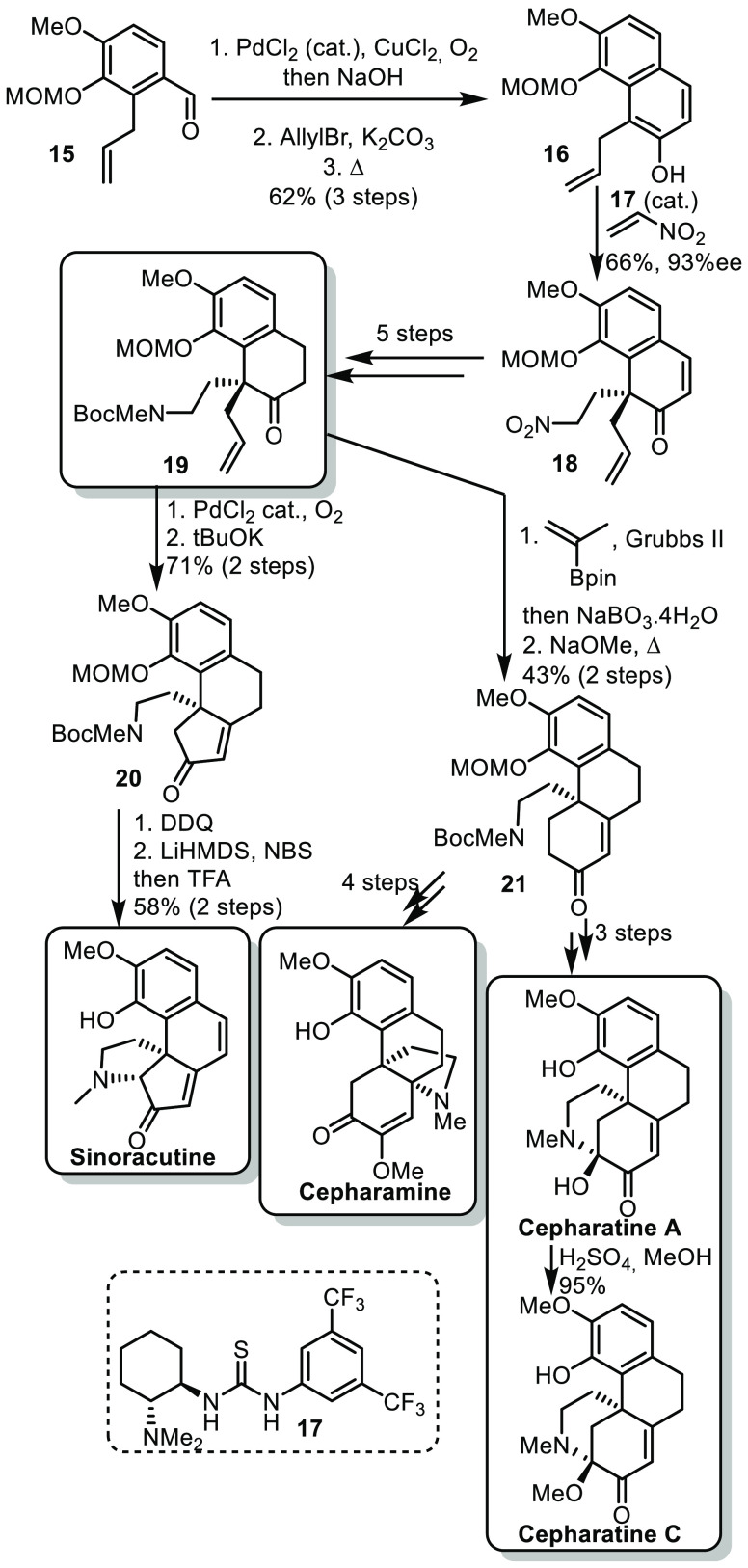
Zhu’s Synthesis of Hasubanan Alkaloids

In 2021, Zhu and co-workers described a strategy for the synthesis of hasubanan alkaloids with diverse skeletons.^20^ The strategy relied on a late formation of the fused or bridged pyrrolidine or piperidine ring from a common intermediate. The synthesis started from known aldehyde **15**, easily accessible in 3 steps from isovanillin.^21^**15** underwent a one-pot Wacker oxidation/aldol condensation, followed by an *O*-allylation and Claisen rearrangement, affording naphthalene derivative **16** (Scheme 4). The naphthol moiety could undergo an enantioselective dearomative alkylation using nitro ethene and Takemoto’s catalyst **17**. The resulting intermediate could undergo a 5 step sequence involving various reductions and protection of the amine group to form the common intermediate **19**. From **19**, a Wacker oxidation and aldol condensation afforded cyclopentenone **20**, from which oxidation and α-bromination/cyclization led to sinoracutine. On the other hand, a cross metathesis using isopropenylboronic acid pinacol ester followed by Brown oxidation and aldol condensation afforded cyclohexanone **21**. The enone could undergo a conjugate addition of the amino group and redox adjustments to complete the synthesis of cepharamine. On the other hand, a series of oxidations, deprotection, and then cyclization afford cepharatine A and C.

In this synthetic sequence, the authors judiciously chose **19** as a common intermediate for both cyclopentenone **20** and cyclohexanone **21**, from which the synthesis of various hasubanan alkaloids could be achieved efficiently. It should be noted that the syntheses relied on similar sequences of aldol condensation, oxidations, desaturation, or deprotection after the point of divergence **19**, which probably contributed to a quick conclusion of this synthesis.

**Scheme 5:**
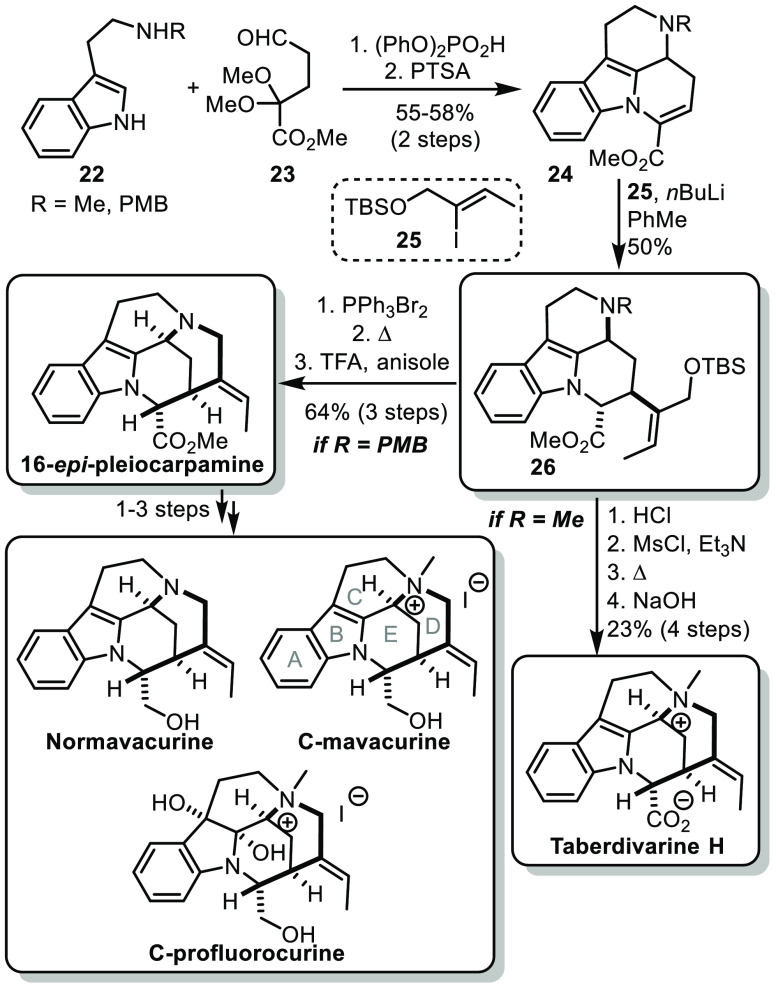
Vincent’s Synthesis of Mavacuran Alkaloids

Following an interest for the mavacuran alkaloid family,^22^ the group of Vincent recently reported a new approach to access the mavacuran skeleton.^23^ Unlike previous approaches, this strategy relies on the late formation of the D ring. For that, the tetracyclic structure bearing rings A, B, C, and E was prepared by a Pictet–Spengler reaction between protected tryptamine **22** and aldehyde **23**, easily obtained in 3 steps from 4-bromobutene (Scheme 5). After acidic treatment, the dimethyl acetal was deprotected and directly condensed with the indole, affording the tetracyclic intermediate **24**. The α,β-unsaturated ester could undergo a 1,4-addition using an organolithium in toluene, an unconventional reactivity that was serendipitously discovered by the authors. The resulting intermediate **26** was the pivotal intermediate of the synthesis. From there, in the case of a PMB-protected amine, the protected primary alcohol could be converted to a primary bromide, which could be substituted by the tertiary amine prior to cleavage of the PMB group, affording 16-*epi*-pleiocarpamine, which could easily be elaborated into normavacurine, C-mavacurine and C-profluorocurine in a few steps. On the other hand, in the case of a simple methylated amine the primary alcohol could be deprotected, mesylated prior to cyclization and saponification of the ester to lead to taberdivarine H.

Overall, through the identification of pivotal intermediate **26** and the late formation of the D ring, the synthesis of various mavacurane alkaloids could be achieved in a remarkably concise manner.

**Scheme 6:**
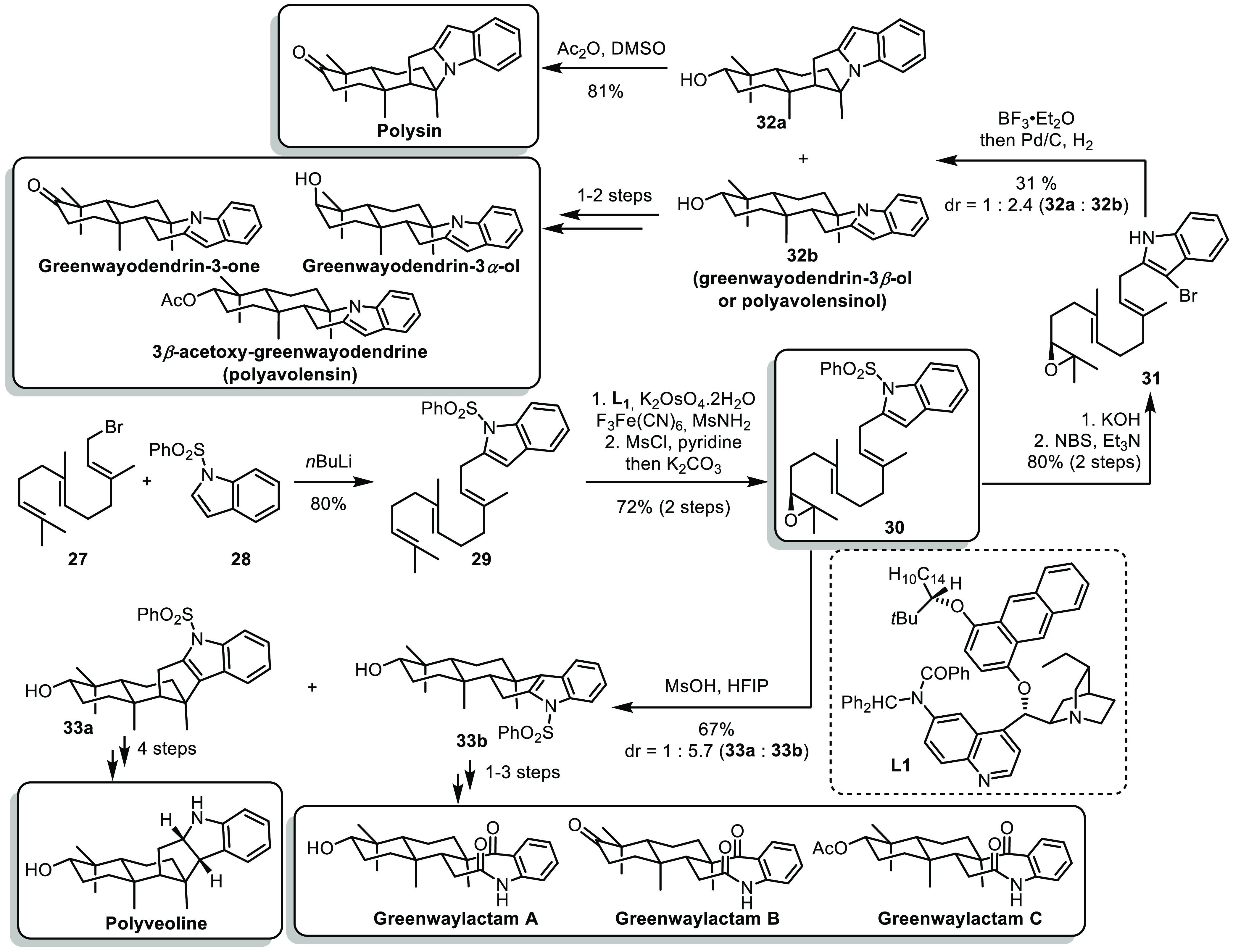
Magauer’s Divergent Synthesis of Sesquiterpene Alkaloids

Later on the same year, the group of Magauer reported a versatile approach for the synthesis of various sesquiterpene alkaloids.^24^ The flexibility of this synthesis is allowed by the development of a divergent polyene cyclization reaction. The precursor for the polyene cyclization was prepared by assembling farnesyl bromide **27** and indole derivative **28** by lithiation/alkylation, enantioselective dihydroxylation and formation of an epoxide **30** (Scheme 6). Deprotection of the benzenesulfonyl group and bromination at C^3^ position of the indole allowed to form **31**, which upon treatment with BF_3_·Et_2_O underwent an uncommon N-terminated polyene cyclization affording a mixture of diastereoisomers **32a** an **32b**. The former could be oxidized into polysin, while the latter was converted into greenwayodendrin-3-one, greenwayodendrin-3α-ol and polyavolensin. On the other hand, **30** could undergo a more classical C-terminated polyene cyclization by treatment with MsOH in HFIP. The resulting diastereoisomers **33a** and **33b** could be further elaborated into polyveoline and greenwaylactams A, B and C.

This work is an excellent example showcasing the power of divergent synthesis: a divergent polyene cyclization cascade was developed, allowing access to two different skeletons from common intermediate **30**, further leading to the synthesis of 9 natural products.

**Scheme 7:**
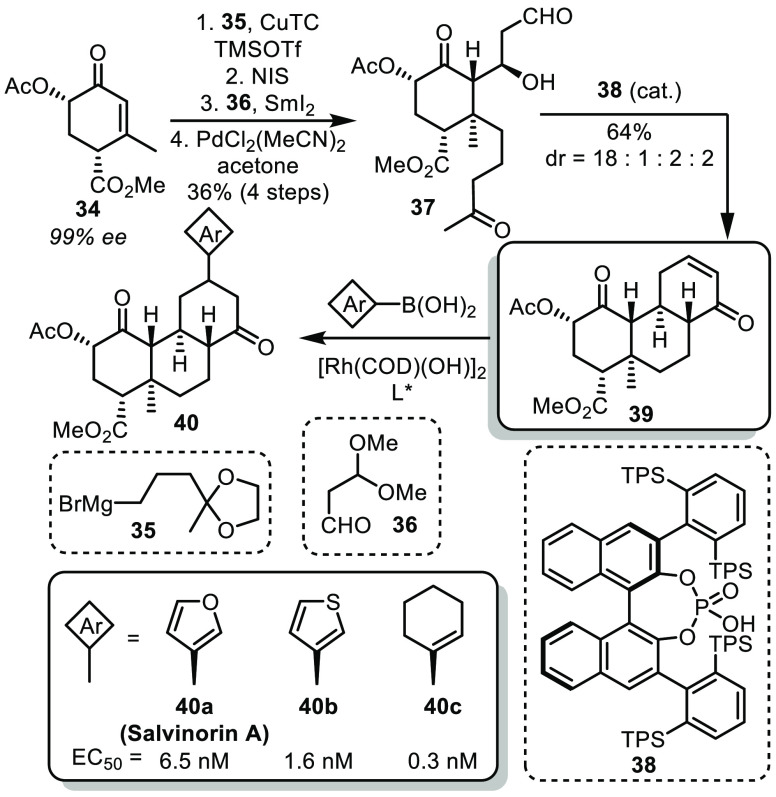
Shenvi’s Synthesis of Salvinorin A Analogs

Beyond the diversity of natural product structures, divergent synthesis can also be applied to reach the chemical space surrounding a specific natural product for medicinal chemistry purposes. In this mindset, Shenvi and co-workers recently reported the synthesis of salvinorin A and various analogs.^25^ 2-Acetoxy-Hagemann’s ester **34**, easily accessible in enantioenriched form in 5 steps from methylvinyl ketone and methyl 2-butynoate, underwent a conjugate addition, α-iodinatio,n and Barbier coupling with malondialdehyde dimethylacetal **36** (Scheme 7). After the cleavage of both acetal protecting groups, keto aldehyde **37** was obtained. This intermediate was subjected to chiral phosphoric acid **38** to trigger a one-pot elimination/diastereoselective Robinson annulation to afford tricyclic enone **39**. From there, Rh-catalyzed conjugate addition using various boronic acids allowed the synthesis of a diversity of salvinorin analogs **40**. Among the synthesized structures, some proved to be more potent than salvinorin A for the inhibition of forskolin-stimulated cAMP accumulation.

The example described above showcases the power of collective synthesis. The key point in this kind of approach is to identify a suitable late intermediate which will bear part of the target’s complexity while allowing access to a diversity of natural products or analogs, as well as an efficient approach to reach this late intermediate.

## Identifying the Right Starting Material: Semisynthesis and Skeletal Editing

Semisynthesis is the specific case in which a molecule is synthesized using a natural product as the starting material.^26^ The flagship example of this approach is docetaxel and paclitaxel semisynthesis from 10-deacetylbaccatin III.^27^ Beyond classical functionalization of natural products, the emergence of C–C bond activation, rearrangements, and other selective C–C cleavages has led to innovative strategies of skeletal editing. Through those, the synthesis of complex structures from abundant natural products with completely different skeletons has been possible. The advantage of these approaches is the possibility to use a starting material already possessing part of the atoms and the complexity of the target molecule.

**Scheme 8:**
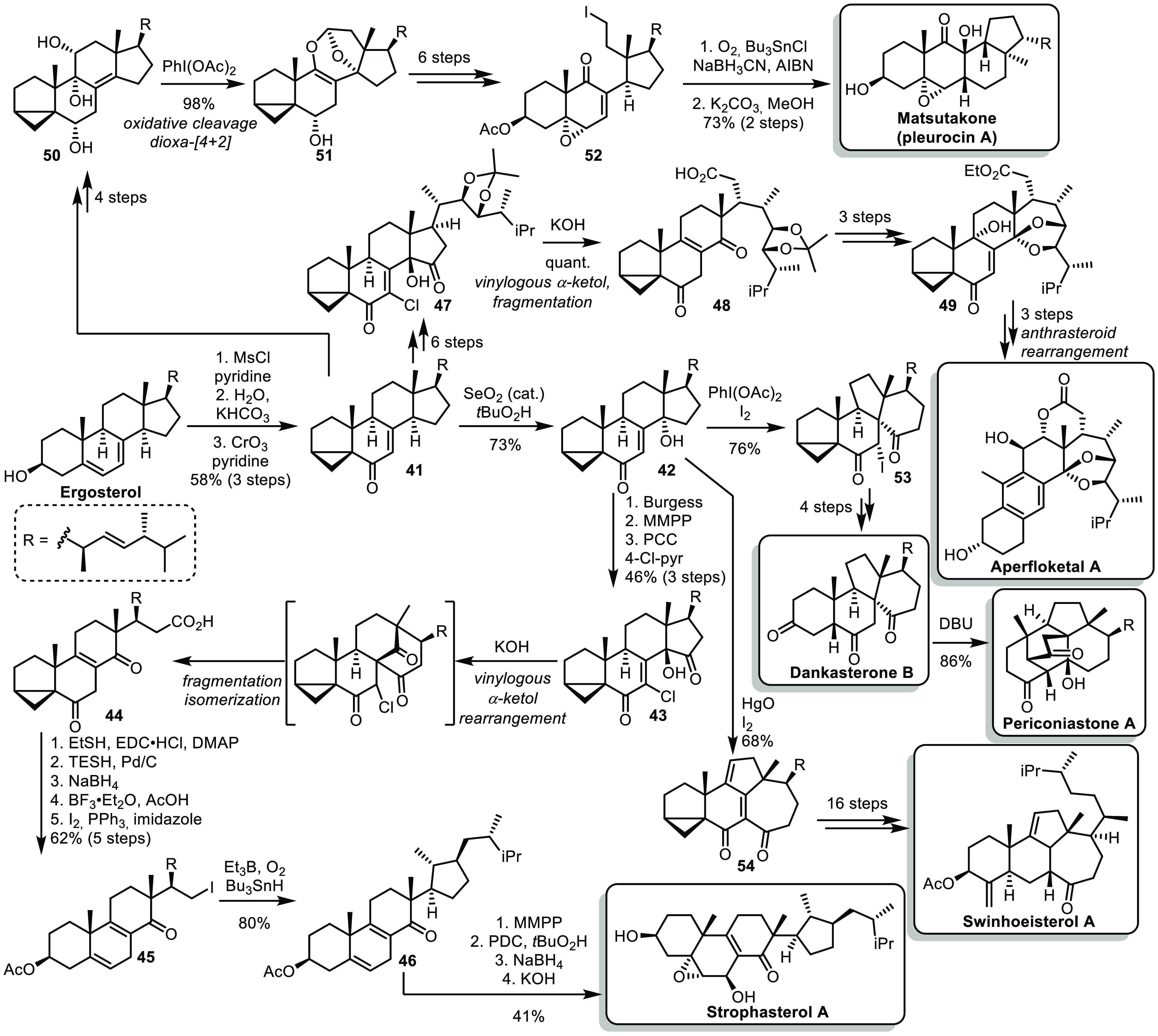
Heretsch’s Syntheses of Steroids by Skeletal Rearrangements from Ergosterol

In recent years, the Heretsch group engaged in a semisynthesis campaign targeting various rearranged steroid structures starting from ergosterol. The authors use the reversible conversion of ergosterol into its analog with fused cyclopentyl/cyclopropyl rings, usually named *i*-steroid^28^ as a way to mask the eastern part of ergosterol while introducing an enone which will serve as chemical handle to further oxidize and edit the western part (Scheme 8).

In 2016, Heretsch described the synthesis of strophasterol A starting from ergosterol.^29^ After conversion into *i*-steroid **41**, an allylic oxidation led to **42**. A series of selective oxidations afforded α-ketol **43** which, upon treatment in basic conditions, underwent a vinylogous α-ketol rearrangement, followed by fragmentation and isomerization. From the resulting intermediate **44**, the carboxylic acid moiety was reduced and iodinated, while the eastern part was regenerated by reduction of the ketone and treatment with BF_3_·Et_2_O and acetic acid. Iodide **45** underwent a reductive radical cyclization to form cyclopentane ring **46**, and further redox ajustments concluded the synthesis of strophasterol A. Later on, the same group applied a similar strategy to the synthesis of aperfloketal A.^30^ A series of selective oxidations from *i*-steroid **41** afforded **47**, from which a similar vinylogous α-ketol rearrangement/fragmentation/isomerization cascade gave **48**. A sequence of esterification, oxidation and deprotection allowed the formation of ketal **49**, which further underwent a 3-step sequence involving an anthrasteroid rearrangement as the key step, leading to aperfloketal A.

In 2019, Heretsch and co-workers used *i*-steroid **41** as an intermediate for the synthesis of matsutakone (pleurocin A).^31^ A series of redox adjustments led to the formation of *vic*-diol **50**, which could undergo an oxidative cleavage, followed by a formal dioxa-[4 + 2] cycloaddition to afford ketal **51**. Further adjustment of the functional groups, including formation of an iodide in place of the ketal and regeneration of the skeleton’s eastern part, led to intermediate **52** bearing a primary iodide and an enone. These would prefigure the key radical cyclization, completing the synthesis of matsutakone.

Finally, in 2020, the same group reported the synthesis of triterpenes with various skeletons.^32^ From intermediate **42**, a one-pot radical 1,2-rearrangement/iodination triggered by PhI(OAc)_2_ and I_2_ afforded intermediate **53**, which could further be elaborated into dankasterone B and periconiastone A. On the other hand, treating the same intermediate with HgO and I_2_ allowed a Dowd–Beckwith rearrangement following the 1,2-rearrangement mentioned previously. The resulting structure **54** led to swinhoesterol A after modification of the R chain, selective reductions, and regeneration of the eastern part.

Overall, this total synthesis program allowed the use of a cheap, abundant, although complex starting material, namely, ergosterol, for the synthesis of various rearranged steroids with diverse skeletons. In particular, the strategies rely on the formation of an *i*-steroid with a key enone moiety which serves as a handle for selective oxidation to further allow skeletal rearrangements.

**Scheme 9:**
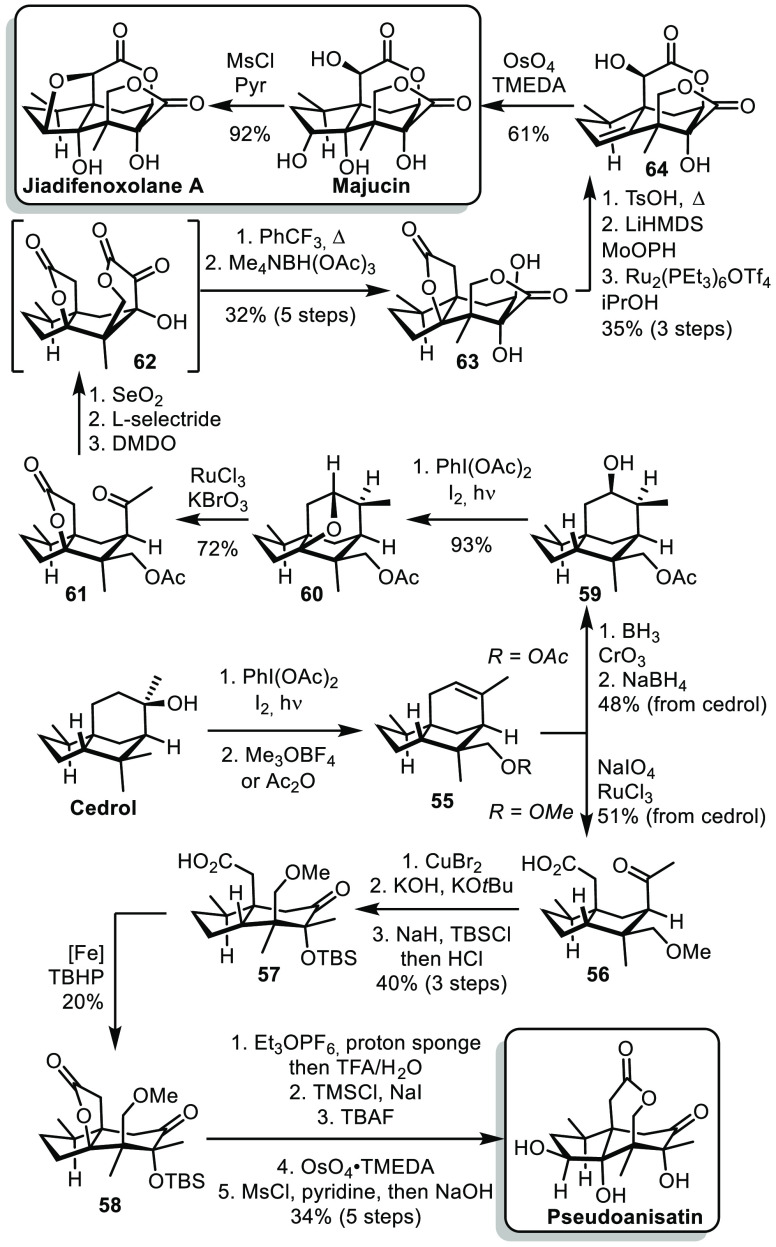
Maimone’s Syntheses of Sesquiterpenoids from Cedrol

During the past decade, the group of Maimone reported the synthesis of various sesquiterpenoids from cedrol. These syntheses rely mainly on meticulous studies of the possible C–H oxidations of the cedrol core.^33^ From cedrol, a Suarez type oxidative cyclization followed by elimination allowed the transfer of the oxygen atom of cedrol onto a remote methyl group (Scheme 9). Oxidative cleavage of the newly formed olefin allowed the formation of **56**.^34^ Oxidation α to the ketone mediated by CuBr_2_ followed be α-ketol rearrangement and protection of the tertiary alcohol afforded **57**. The acid moiety of **57** could direct iron-mediated C–H oxidation to form lactone **58**. Finally, a sequence of elimination, deprotection, lactonization, directed dihydroxylation, and inversion of the resulting secondary alcohol afforded pseudoanisatin. On the other hand, from **55**, a hydroboration led to secondary alcohol **59** after a sequence of oxidation/reduction necessary to obtain the correct relative configuration.^35^ Again, a Suarez-type oxidative cyclization allowed the formation of bridged THF structure **60**. Treatment with *in situ* prepared RuO_4_ allowed remarkable C–C bond cleavage. After a series of redox adjustments and lactonization, the intermediate lactone **62** bearing an α-ketol moiety was obtained. The use of DMDO was crucial for this last oxidation to obtain the desired relative configuration. Intermediate **62** was unstable and could undergo an α-ketol rearrangement. After reduction, the tetracyclic structure **63** was obtained. Acid-mediated elimination of the propellalactone, lactonization, α-hydroxylation, and inversion of configuration of the newly form hydroxyl substituent afforded **64**. Finally, directed hydroxylation led to the formation of majucin, which could further undergo a substitution reaction to form the bridged THF structure of jiadifenoxolane A.

**Scheme 10:**
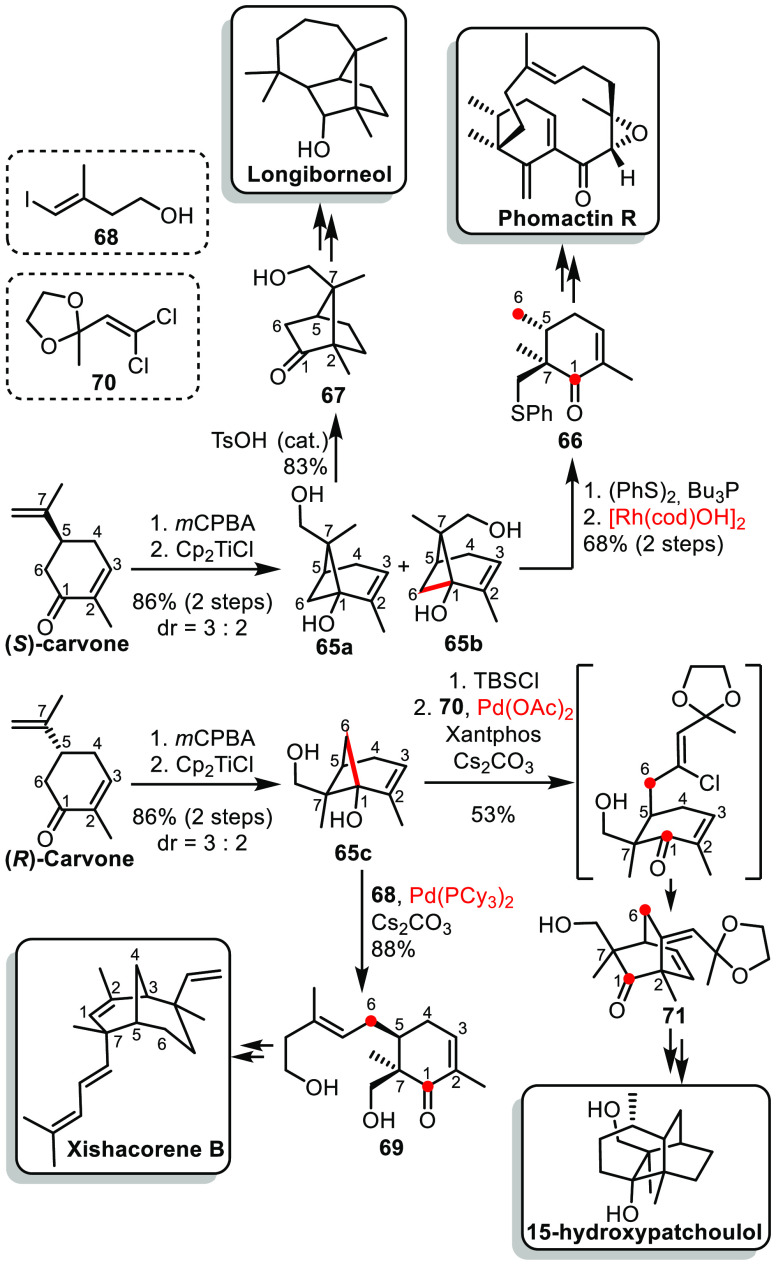
Sarpong’s Skeletal Editing from Carvone

An impressive skeletal remodeling, although starting from a more classical terpene building block, carvone, was described by Sarpong and co-workers and applied to various natural products.^36^ The isopropylene moiety could undergo an epoxidation, followed by Ti^III^-mediated reductive cyclization (Scheme 10).^37^ The resulting synthetic pinene derivative **65** was isolated as a mixture of two separable diastereoisomers. From the minor diastereoisomer **65b**, the primary alcohol could be converted into a sulfide, prior to a Rh-catalyzed C–C activation to cleave the C^1^–C^6^ bond, leading to a rearranged cyclohexene scaffold **66**. This could further be elaborated into various members of the phomactin family.^38^ On the other hand, the major isomer **65a** could undergo an acid-catalyzed semipinacol rearrangement to form camphor-analog **67**, which could also be converted into various members of the longiborneol sesquiterpenoid family.^39^ Starting from (*R*)-carvone, the same sequence of epoxidation/reductive cyclization led to **65c** as the major isomer. From this pinene derivative, a Pd-catalyzed C^1^–C^7^ activation followed by cross-coupling with alkenyl iodide **68** led to the formation of polysubstituted cyclohexanone **69**.^40^ This intermediate later led to the synthesis of xishacorene B via a key transannular Giese cyclization to form the bridged structure. Finally, synthetic pinene derivative **65c** could also be subjected to a Pd-catalyzed C–C activation/cross-coupling/Mizoroki–Heck cyclization with 1,1-dichloro alkene **70**.^41^ The resulting bicyclo[2.2.2]octane was a key intermediate in the synthesis of 14- and 15-hydroxypatchoulol.

**Scheme 11:**
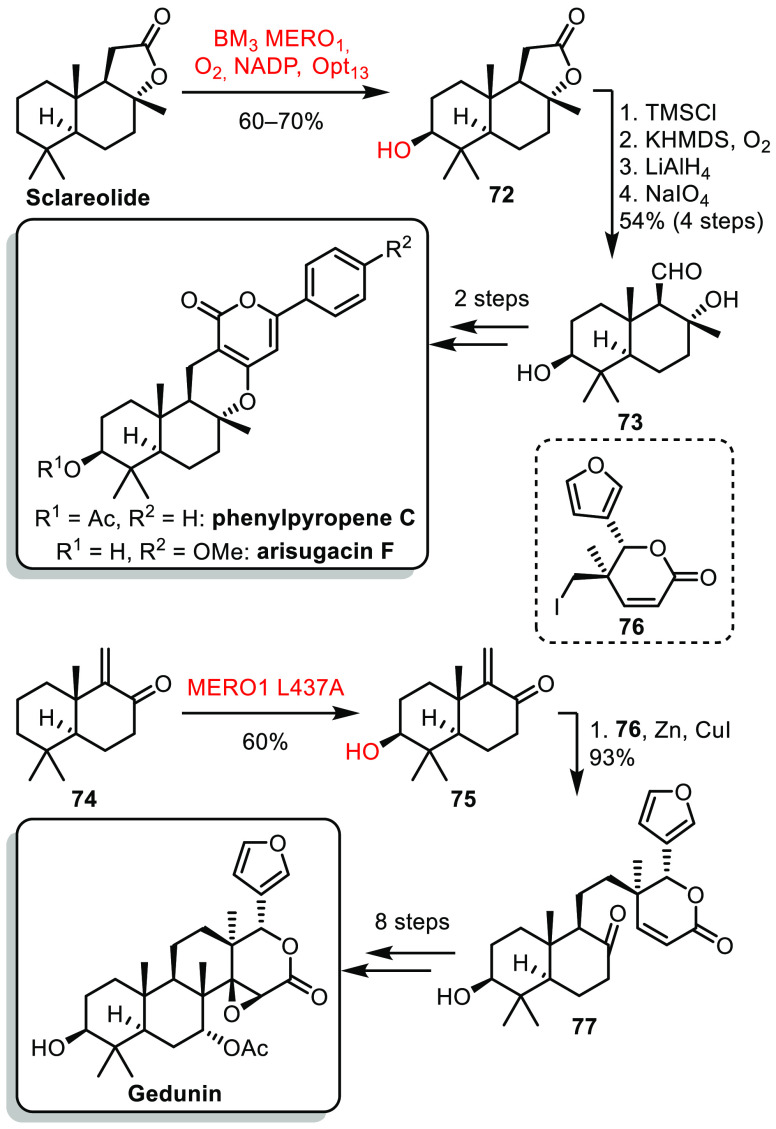
Renata’s Selective Hydroxylation of Sclareolide Derivatives

Directed evolution offers new exciting perspectives for the formation of complex structures.^42^ In 2020, the group of Renata reported the selective C^3^ hydroxylation of sesquiterpene lactone sclareolide using a P450_BM3_ variant.^43^ From this cheap and abundant starting material, added value hydroxylated product **72** could be obtained efficiently on gram-scale (Scheme 11). Protection of the secondary alcohol, hydroxylation to the lactone, reduction, and oxidative cleavage allowed the formation of **73** bearing an aldehyde and secondary alcohol as chemical handles for the construction of meroterpenoids phenylpyropene C and arisugacin F. Although very powerful, the use of this P450_BM3_ variant was limited to few substrates. The same authors had to develop a new variant to perform efficiently the selective oxidation of **74**, easily obtained in three steps from sclareolide.^44^ The resulting intermediate **75** could be coupled with iodide **76** through a zincation/conjugate addition. The resulting advanced intermediate **77** was then converted to gedunin in an 8-step sequence.

These selected examples highlight the importance of finding the right starting material in synthesis. Starting from cheap, abundant, although complex structures such as sclareolide or ergosterol, the synthesis of various complex natural products could be achieved very efficiently.

## Identifying the Right Key Step(s) For Concise Syntheses

The design of a synthetic strategy uses retrosynthetic analysis, the rules of which were established decades ago by Corey. The idea is to identify key disconnections to make the synthesis as concise and efficient as possible.^45^ In recent years, the rise of AI and computer assistance allowed to simplify the reflection necessary to set up a strategy and opened new avenues for organic synthesis, especially using the software Chematica.^46^ Nevertheless, various recent examples can be cited to show that an old-fashion blackboard design of a retrosynthesis can still lead to success.

In 2016, the group of Maimone reported an efficient synthesis of 6-*epi*-ophiobolin N.^47^ The ophiobolins are a family of complex sesterterpenoids displaying a 5–8–5 fused ring system with many stereogenic centers. Maimone’s hypothesis was that the tetracyclic core could be constructed by radical cyclization. This approach would have two main advantages: (i) the substrate for this key cyclization could be easily accessed from common terpenic precursors such as farnesol and linalool and (ii) after the key cyclization all the stereogenic centers would be set and most functional groups would be at hand’s reach.

**Scheme 12:**
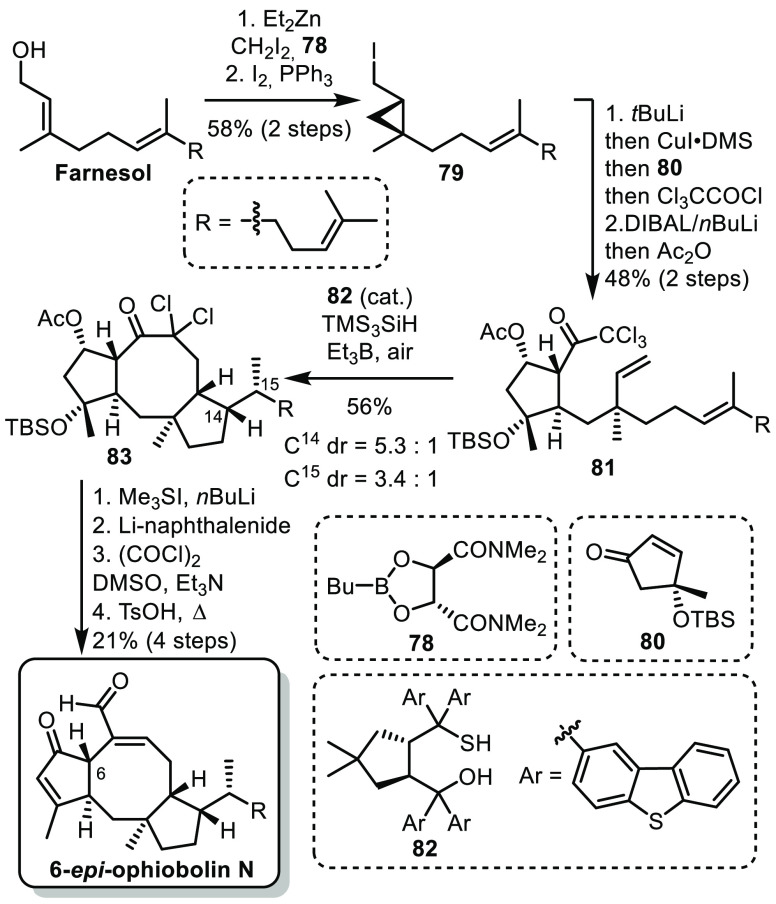
Maimone’s Synthesis of 6-*epi*-Ophiobolin N

The synthesis began by an enantioselective Charette cyclopropanation of farnesol followed by iodination to form **79** (Scheme 12). This was assembled with precursor **80** (easily prepared in 3 steps from linalool) by a one-pot lithiation/transmetalation/conjugate addition/trapping with trichloroacetyl chloride. After reduction of the ketone and protection, a precursor for the key radical cyclization **81** was obtained. The key cyclization was achieved using a chiral thiol catalyst **82** developed for this purpose, Et_3_B and air as the radical initiator and TMS_3_SiH as the hydrogen radical source. The tricyclic structure **83** was obtained as a mixture of diastereoisomers at C^14^ and C^15^, which could be separated in later stages of the synthesis. From **83**, a sequence of Corey-Chaykovsky epoxidation, reductive opening of the expoxide, Swern oxidation and elimination of the tertiary alcohol concluded the synthesis of 6-*epi*-ophiobolin N in 10 steps (longest linear sequence). A few years later, the same authors applied a similar strategy to the synthesis of 6-*epi*-ophiobolin A.^48^

Overall, this work by Maimone led to a very efficient synthesis of the C^6^-epimer of natural ophiobolin N in only 10 steps. This was possible thanks to the identification and development of a key radical cyclization to form the tricyclic structure but also to a smart experimental design in which many concession steps are performed in the same pot as constructive steps.

**Scheme 13:**
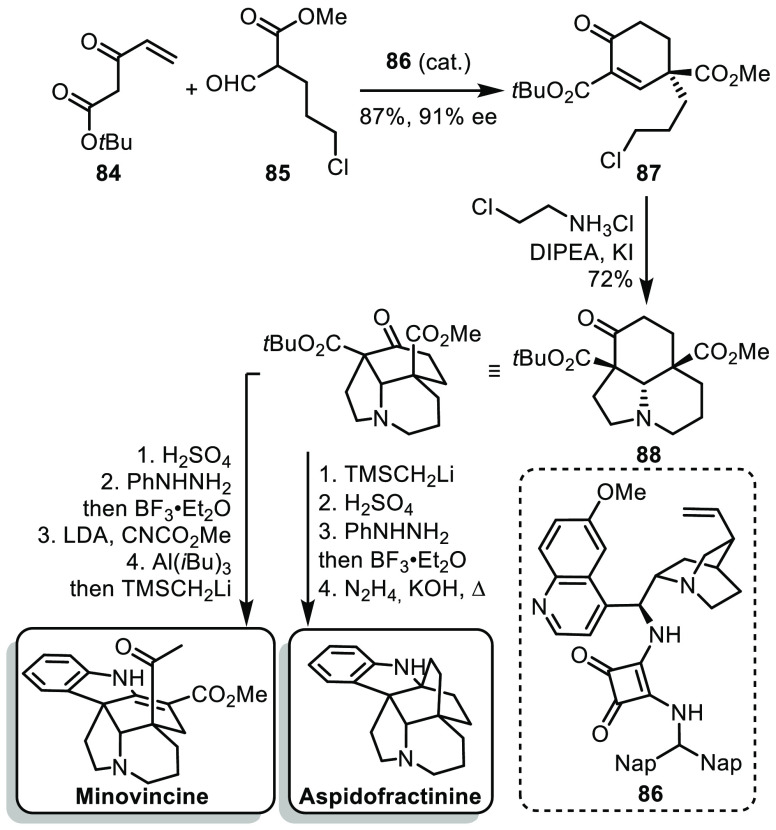
Soós’ Synthesis of Minovincine and Aspidofractinine

In 2020, the group of Soós reported an expeditious synthesis of minovincine and aspidofractinine.^49^ The synthesis relied on quick access to a tricyclic core, followed by late formation of the indole moiety. Enone **84** and malonate derivative **85**, respectively prepared in 2 and 1 step from commercial precursors, where assembled by an enantioselective Robinson annulation using organocatalyst **86** (Scheme 13). The reaction afforded cyclohexenone **87** in a good yield and excellent enantioselectivity. Cyclohexenone **87** underwent a cascade of nucleophilic substitutions and conjugate addition in the presence of 2-chloroethylamine, affording the tricyclic core **88**. Remarkably, this intermediate could be prepared on a 59 g scale. From there, a sequence of decarboxylation, Fischer indolization, methoxycarbonylation, and selective ketone formation led to the formation of minovincine. On the other hand, starting by the methyl ketone formation followed by decarboxylation allowed an interrupted Fischer indolization to build the bridged structure of aspidofractine. The synthesis was concluded by a Wolff–Kishner reduction of the bridged ketone.

In this work, Soós and co-workers achieved a straightforward synthesis of minovincine and aspidofractinine in 8 steps. This was made possible by the quick formation of a tricyclic core using an organocatalytic Robinson annulation and a nucleophilic cascade. The power of this synthesis was showcased by the synthesis of more than 1 g of minovincine.

**Scheme 14:**
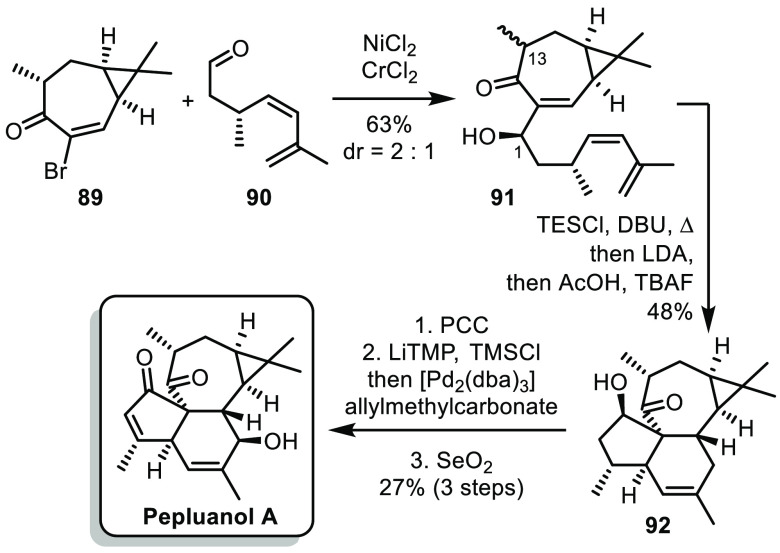
Gaich’s Synthesis of Pepluanol A

In 2021, the group of Gaich tackled the synthesis of pepluanol A using a key intramolecular Diels–Alder cycloaddition (IMDA).^50^ The synthesis used bicyclic precursor **89**, easily prepared in 5 steps from carene using a procedure previously described by Baran,^51^ and aldehyde **90**, prepared in 5 steps from (*S*)-3-bromo-2-methylpropanol (Scheme 14). These fragments were assembled by a Nozaki–Hiyama–Kishi reaction, affording a mixture of diastereoisomers at C^1^ and C^13^. While the minor diastereoisomer at C^1^ could be separated by column chromatography, separating the C^13^-epimers proved to be irrelevant. Indeed, after TES protection of the secondary alcohol, the IMDA reaction proceeded in a diastereoconvergent fashion in the presence of DBU. Nevertheless, the product had the undesired configuration at C^13^ and the authors had to epimerize it *in situ* by treatment with LDA. The tetracyclic intermediate **92** was then converted into pepluanol A by a sequence of 3 selective oxidations. Thus, the synthesis of pepluanol A was achieved in 10 steps from commercially available precursors.

**Scheme 15:**
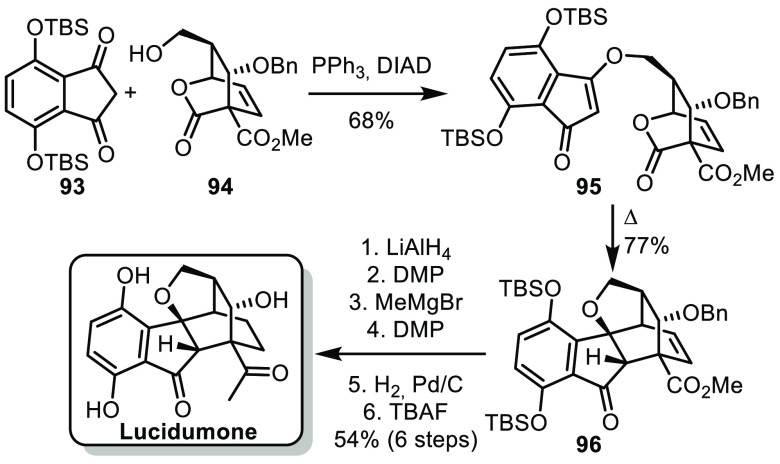
de la Torre’s Synthesis of Lucidumone

In 2022, our group reported the first enantioselective synthesis of lucidumone.^52^ Our strategy relied on a quick assembly of the pentacyclic scaffold using a retro-[4 + 2]/CO_2_ extrusion/IMDA cascade. The precursor for this key step was synthesized from indanedione **93** and bridged bicyclic lactone **94**, respectively, synthesized in 4 and 5 steps from commercially available material (Scheme 15). In particular, **94** was made enantioselectively using an inverse electron demand Diels–Alder cycloaddition involving a 2-pyrone.^53,54^ The fragments were assembled under Mitsunobu conditions to form **95**, from which the key retro-[4 + 2]/CO_2_ extrusion/IMDA cascade afforded **96** with the pentacyclic structure of lucidumone. A six-step sequence allowed conversion of the methyl ester into a methyl ketone, reduction of the endocyclic olefin, and cleavage of the protecting groups to conclude this synthesis in 13 steps (longest linear sequence). Remarkably, 1.6 g of the natural product was synthesized in one batch by using this approach.

**Scheme 16:**
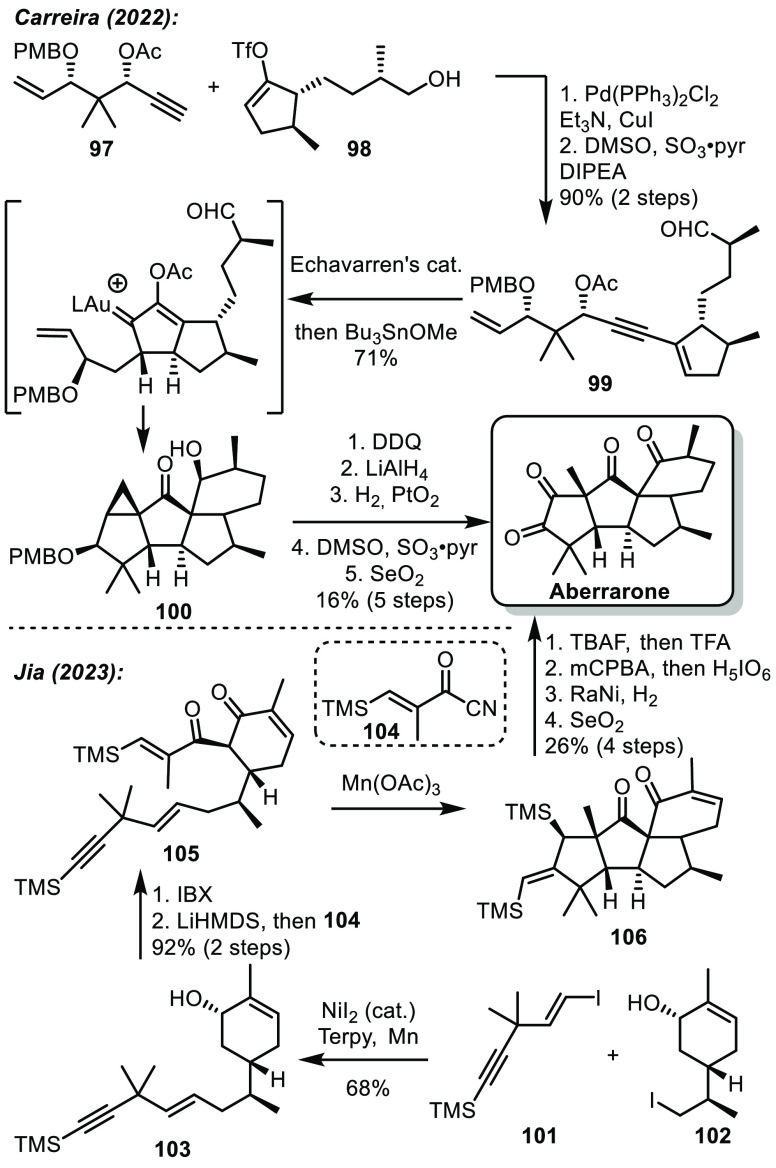
Carreira and Jia’s Syntheses of Aberrarone

In 2022, the team of Carreira reported an elegant synthesis of aberrarone.^55^ In particular, Carreira’s approach features an impressive Au-catalyzed cyclization cascade based on previous studies by Fensterbank and co-workers.^56^ Precursors **97** and **98**, easily obtained in 7 steps from pantolactone and Roche ester respectively, were assembled by a Sonogashira coupling (Scheme 16). After oxidation of the primary alcohol, precursor **99** was obtained. This could undergo the key Meyer–Schuster/Nazarov cascade, followed by an intramolecular cyclopropanation and aldol reaction to reach the skeleton of aberrarone **100**. From there, redox adjustments allowed completion of the synthesis in 15 steps.

A few months later, the group of Jia reported a different synthesis of the same natural product.^57^ Jia’s approach involved intermediates **101** and **102**, synthesized in 2 steps from commercial compounds. The fragments were assembled by Ni-catalyzed reductive cross coupling to afford **103**. Oxidation of the allylic alcohol and α-acylation allowed the formation of **105**, the precursor for the key cyclization. After treatment with Mn(OAc)_3_, **105** underwent a radical cascade leading to the tetracyclic core of aberrarone **106**. Final redox adjustments concluded this synthesis in 12 steps.

While both approaches employ an impressive cascade cyclization to form the tetracyclic structure, Jia’s approach proved to be slightly shorter. The main advantage of this second approach lies in the quick formation of fragments **101** and **102**.

**Scheme 17:**
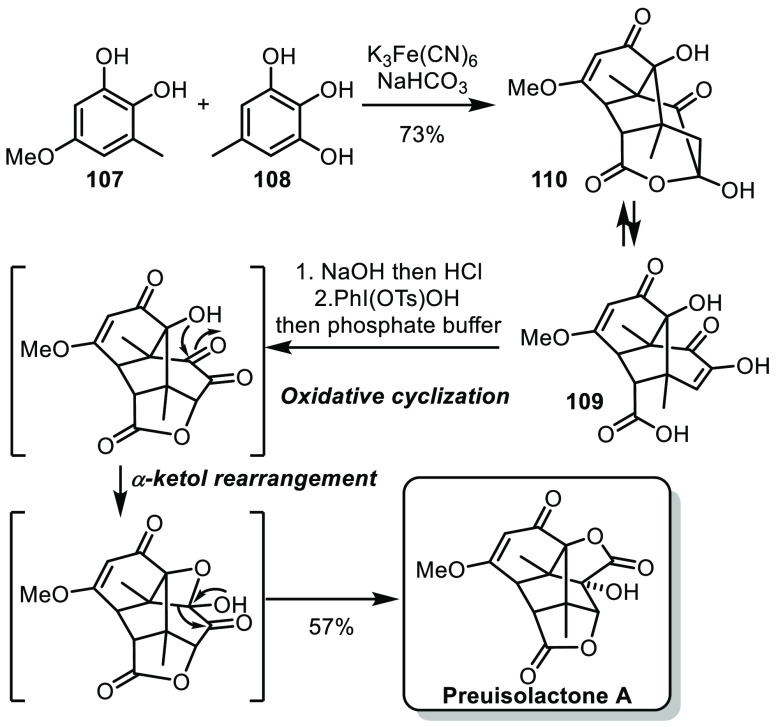
Trauner’s Biomimetic Synthesis of Preuisolactone A

In an effort to find the shortest possible synthesis, biosynthetic considerations are often very valuable.^58^ In this context, Trauner and co-workers recently reported an impressive biomimetic synthesis of preuisolactone A produced by the endophytic fungus *Preussia isomera*.^59^ Trauner’s hypothesis was that preuisolactone A was formed in the plant from catechol **107** and pyrogallol **108** by oxidation/(5 + 2)-cycloaddition followed by a series of cyclizations, fragmentations, and rearrangements. With this in mind, the authors treated precursors **107** and **108** in oxidative conditions and obtained the bridged tricyclic intermediate **109** in equilibrium with its lactol form **110** (Scheme 17). The equilibrium could be displaced toward **109** by treatment in basic conditions followed by acidic workup. Treatment with Koser’s reagent allowed an oxidative cyclization, followed by a cascade lactol formation/α-ketol rearrangement, affording preuisolactone A in 57%. This very straightforward synthesis (3 steps) could support the author’s biosynthetic proposal.

**Scheme 18:**
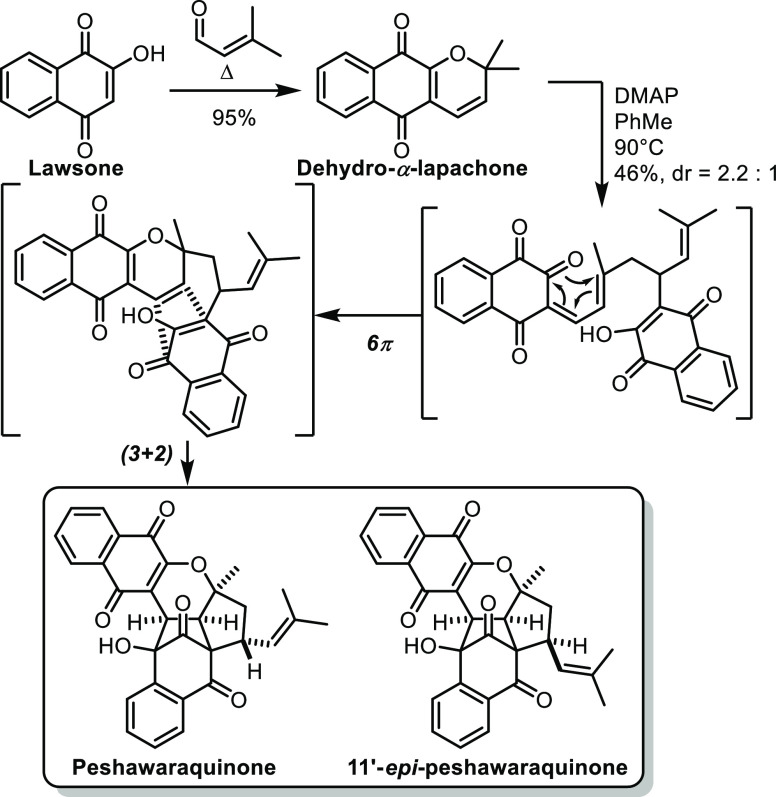
George and Lawrence’s Synthesis of Peshawaraquinone

With a similar mindset, the groups of George and Lawrence recently studied the biosynthesis of peshawaraquinone, a complex polycyclic meroterpenoid isolated from the tree *Fernandoa adenophylla*.^60^ The hypothesis was that peshawaraquinone was formed by unsymmetrical dimerization starting from dehydro-α-lapachone, a meroterpenoid commonly encountered in these trees. To prove this, the authors first synthesized dehydro-α-lapachone from lawsone by a one-pot Knoevenagel condensation and subsequent oxa-6π-electrocyclization (Scheme 18). Then, dehydro-α-lapachone was treated with DMAP in toluene. After 6π-electrocyclic ring opening and intermolecular Michael addition, followed by 6π electrocyclization and intramolecular (3 + 2)-cycloaddition, peshawaraquinone was obtained along with its C^11’^ epimer. Interestingly, when DMAP was replaced with DIPEA, the diastereomeric ratio was reversed. This one-step synthesis of peshawaraquinone from dehydro-α-lapachone could back the author’s biosynthetic proposal, while providing a convenient approach in the lab.

Overall, in this final section, we selected various examples of old-fashioned retrosynthetic design leading to particularly efficient syntheses. A key lesson to take from this collection is the importance of cascade reaction and cycloadditions, or more generally reactions involving multiple bond formation. Through the identification of such key steps, the authors are often able to achieve concise syntheses (even allowing the use of various “concession steps”).

## Conclusion

The field of total synthesis still has a rosy future. In this Perspective, we have highlighted selected recent achievements in this field. We have tentatively organized them depending on the key strategic question that was tackled in each of these approaches. Some of them dealt with flexibility, through the identification of a key intermediate allowing access to a diversity of targets in the context of collective synthesis. Others blew a wind of change on semisynthesis, offering unexpected and exciting skeletal remodeling to reach complex targets from already complex but abundant starting materials. Finally, some of them were about simplicity: finding the right disconnection in a classical retrosynthetic design to allow concise approaches toward complex structures. While every synthesis work described above has its own strong point, none of them is perfect. This perpetual search for perfection is what brings the field of organic synthesis forward and the reason why it still has good days ahead.

## Data Availability

The data underlying this study are available in the published article.

## Abbreviations

AIBN: azobis(isobutyronitrile)
cAMP: cyclic adenosine monophosphate
COD: cyclooctadiene
DBU: 1,8-diazabicyclo[5.4.0]undec-7-ene
DDQ: 2,3-dichloro-5,6-dicyano-1,4-benzoquinone
DIAD: diisopropyl azodicarboxylate
DIPEA: *N*,*N*-diisopropylethylamine
DMAP: 4-dimethylaminopyridine
DMDO: dimethyldioxirane
DMS: dimethylsulfide
DMSO: dimethyl sulfoxide
EDC: 1-ethyl-3-(3-(dimethylamino)propyl)carbodiimide
HFIP: hexafluoroisopropanol
HMPA: hexamethylphosphoramide
IBX: 2-iodoxybenzoic acid
IMDA: intramolecular Diels–Alder
LDA: lithium diisopropylamide
*m*CPBA: *meta*-chloroperbenzoic acid
MMPP: magnesium monoperoxyphthalate
MOM: methoxymethyl
NIS: *N*-iodosuccinimide
PCC: pyridinium chlorochromate
PDC: pyridinium dichromate
PMB: *para*-methoxybenzyl
PTSA: *para*-toluenesulfonic acid
TBDPS: *tert*-butyldiphenylsilyl
TBS: *tert*-butyldimethylsilyl
TES: triethylsilyl
TFA: trifluoroacetic acid
THF: tetrahydrofuran
TMEDA: tetramethylethylenediamine
TMS: trimethylsilyl
TPS: triphenylsilyl
